# Longitudinal Follow Up of Early Career Midwives: Insights Related to Racism Show the Need for Increased Commitment to Cultural Safety in Aboriginal Maternity Care

**DOI:** 10.3390/ijerph18031276

**Published:** 2021-01-31

**Authors:** Rosalie D. Thackrah, Jennifer Wood, Sandra C. Thompson

**Affiliations:** 1 Western Australian Centre for Rural Health, University of Western Australia, Nedlands 6009, Australia; sandra.thompson@uwa.edu.au; 2 Labour and Birth Suite, Maternal Fetal Assessment Unit, King Edward Memorial Hospital, Subiaco 6008, Australia; Jennifer.Wood@health.wa.gov.au

**Keywords:** Aboriginal maternal health, midwifery education, longitudinal follow up, casual racism, institutional racism, cultural safety in maternity care

## Abstract

Racism in health care undermines equitable service delivery, contributes to poorer health outcomes and has a detrimental effect on the Aboriginal workforce. In maternity care settings, Aboriginal women’s perceptions of discrimination are widespread, with the importance of cultural practices surrounding childbirth often not recognised. Efforts to build midwives’ cultural capabilities and address health disparities have seen Aboriginal content included in training programs but little is known about its application to clinical practice. This study reinterviewed midwives who had previously completed university midwifery training that aimed to increase understanding of Aboriginal people and cultural safety in health care. Participants were 14 non-Indigenous midwives and two Aboriginal midwives. Interviews explored the legacy of program initiatives on cultural capabilities and observations and experiences of racism in maternity care settings. Methods followed qualitative approaches for research rigour, with thematic analysis of transcribed interviews. Findings revealed the positive impact of well-designed content and placements, with non-Indigenous participants cognisant and responsive to casual racism but largely not recognising institutional racism. The Aboriginal midwives had experienced and were attuned to racism in all its guises and suggested initiatives to heighten awareness and dispel stereotypes. It is evident that greater attention must be paid to institutional racism in educational programs to increase its recognition and appropriate actions within health care settings.

## 1. Introduction 

The corrosive effects of racism on the physical and emotional well-being of Aboriginal and Torres Strait Islanders (hereafter called ‘Aboriginal’ or ‘Indigenous Australians’) have been widely documented over the last two decades, with racism identified as an important determinant of health. Numerous studies of self-reported racism emerged in the early 2000s in response to a paucity of data on health impacts; these revealed the physical and emotional toll exacted on Aboriginal people of all ages due to racist behaviours perpetrated and widely tolerated in the broader community [1,2,3,4,5]. An international systematic review on racism and health, which included Australian studies, observed that “the most consistent relationship between self-reported racism and health was found for negative mental health outcomes” [3] (p. 892). While mixed results were observed regarding physical health impacts, a lag between exposure to racism and the development of physical health problems may contribute to these findings, especially in studies using cross-sectional designs [3,6]. Experiences of racism have also been associated with underutilisation of mainstream health care services, delays in seeking care, mistrust of providers and failure to follow recommendations, all contributors to poorer health outcomes for Aboriginal Australians [7]. Furthermore, racism has a detrimental effect on the recruitment and retention of the Aboriginal health workforce [7].

Racism has been defined as “organized systems within societies that cause avoidable and unfair inequalities in power, resources, capacities and opportunities across racial or ethnic groups” [4] (p.1). Racism is a social construct concerned with the effect of people’s actions on others, regardless of their intentions and is expressed through stereotypes, prejudices or discriminatory behaviour [2,8]. It is a form of oppression, which can be logically twinned with its opposite, the notion of privilege and is formulated around social and cultural characteristics that highlight innate differences between groups [2]. This culminates in a process of “othering” whereby self-identity is strengthened through attributing different and often negative characteristics to others [9].

Racism can arise in interpersonal relationships, organisational settings or be internalised in individuals who experience discrimination based on race [1,3]. Racism may be overt, but often is subtle. Casual racism and implicit or unconscious bias are frequently used terms coined to capture disparaging comments that are based on stereotypes or prejudice; these comments may not be intended to cause harm or offence but have an impact regardless of intent [10,11]. Shirodkar’s recent study of the extent of implicit bias towards Indigenous Australians found that close to 75% displayed this tendency, although the impact on health outcomes was not explored [12]. Fitzgerald and Hurst’s systematic review of implicit bias among health care professionals revealed that biases are likely to influence diagnosis and treatment decisions and they recommended further investigation into health outcomes [11].

The concept of institutional racism was coined more than 50 years ago and focuses on organisational policies and procedures that increase power differentials between racial groups [13]. While some still see institutional racism as the sum total of employees’ behaviours, for others it arises out of “the policies, or structure of an institution in the absence of racist individuals” [13] (p. 612). Bourke and colleagues viewed intent as immaterial; the effect of institutional racism is that it contributes to poorer health outcomes for Aboriginal people. They noted that cultural education and anti-racist programs are unlikely to address institutional racism given their focus on individual attitudes and behaviours, rather than on the structures that generate racial disparities. Instead, they suggest that efforts to ameliorate institutional racism require practitioners to “recognise the key indicator of poorer health outcomes, and to then seek change within their hospital or healthcare organisation” [13] (p. 611).

Poorer health outcomes for Indigenous Australians compared with the wider community are well established, with many chronic diseases having their genesis in pregnancy and early life [14]. Birth outcomes for Aboriginal women, especially higher rates of low birth weight, preterm birth and infant mortality are known contributors to future health disparities; addressing these outcomes and associated risk factors to reduce disparities remain a “Close the Gap” target [14,15]. Socio-economic disadvantage, risky health behaviours and slow progress in the provision of culturally safe maternity services, especially to rural and remote women, are all implicated in poorer health outcomes and have their roots in Australia’s colonial history. European colonisation of Australia in the late 18th century resulted in the dispossession and dislocation of Aboriginal populations. The introduction of new diseases, grazing animals, alcohol and paternalistic policies dramatically reduced numbers and eroded cultural identity [16]. Government policies intended to ‘protect’ Aboriginal people from frontier violence and new diseases included forced removal from traditional lands to Christian missions and government settlements [16]. This was followed in the mid-19th century by the removal of so called ‘half-caste’ children from families (the Stolen Generation). Deemed as an ‘absorption’ policy, it was not dismantled until the 1970s [17]. The physical and emotional health of those who survived this violence, mistreatment and discrimination were severely diminished and intergenerational trauma continues to be felt and evident today.

The National Apology to Australia’s Indigenous Peoples delivered in 2008 by then Prime Minister Kevin Rudd was one of a number of initiatives which recognised the impact of past traumas due to government policies and aimed to promote healing [18]. The ‘Close the Gap’ campaign, which commenced a year earlier, highlighted ongoing Indigenous disparities in health, education, employment and incarceration rates, and drew attention to service delivery inadequacies; it also set targets for improvement. Australia’s National Maternity Services Plan (NMSP) was developed in this context and in the knowledge that cultural respect in maternity care provision is essential to improved health outcomes [19]. It identified priority areas targeting maternal health disparities; these include an expansion of the Indigenous maternity workforce, the provision of culturally competent maternity care and dedicated programs for ‘birthing on country’ (‘birthing on country’ refers to giving birth on the land of an Aboriginal woman’s own birth or on the land of the father of the child.) [14].

Recognition that maternity care experiences are associated with health outcomes and a potential barrier to future health care utilisation has led to a closer examination of Aboriginal women’s birthing stories on Noongar Boodjar lands, the ancestral country of the Noongar people of the south-west of Western Australia. Noongar culture has been described as collectivist in character and female members of the extended family, especially aunties and grandmothers, often play an important role in maternity care [20]. Traditionally, they assisted during birthing and were closely involved in their grandchild’s spiritual and emotional development. Today, their role as a conduit for intergenerational knowledge and as a support person for the new mother remains significant in many families [20]. The recent ‘Birthing on Noongar Boodjar’ study explored four generations of Aboriginal women’s stories and experiences of childbearing [21]. The study revealed the on-going significance of cultural practices surrounding childbirth including the support of extended family, intergenerational knowledge sharing, and enhanced cultural security provided by Aboriginal staff [21]. It was observed that maternity care providers often do not recognise or support Aboriginal cultural practices associated with childbirth and without this safety net, women often feel alone and alienated. Both the inflexibility of maternity services and incidents of racism where women felt “undermined and very small” were associated with negative care experiences [21] (p. 398).

Another study conducted in South Australia which explored the relationship between perceived discrimination in perinatal care and birth outcomes among 344 Aboriginal women reported that 51% had experienced discrimination or unfair treatment, and that these women had poorer birth outcomes including lower-birth-weight babies [22]. These findings suggest that racism continues to be experienced by pregnant and birthing Aboriginal women and provide strong support for the NMSP’s priority areas aimed at improving Aboriginal maternity services, and including cultural competence in training programs.

Australian medical and health professional accreditation bodies require Aboriginal content to be embedded in training programs, and curriculum frameworks have been developed to support implementation [23,24]. Despite this, the amount, nature and delivery of content varies considerably. While encouraging signs have emerged about the immediate impact of Aboriginal content on students’ developing cultural capabilities [25,26,27,28], few long-term evaluations have been conducted into the sustainability of these gains and the extent to which they are applied in workplaces [8,27]. Furthermore, if content is limited to an introductory unit, it is unlikely that the complex issues surrounding racism and its health impacts will be addressed in sufficient depth. Yet, a key focus of the recently released National Scheme’s Aboriginal and Torres Strait Islander Health and Cultural Safety Strategy 2020–2025 for health practitioners is the persistence of racism in the health care system and the urgent need to eliminate it [29]. Culturally safe practice is determined by Aboriginal people and defined as “the ongoing critical reflection of health practitioner knowledge, skills, attitudes, practising behaviours and power differentials in delivering safe, accessible, responsive healthcare free of racism” [29] (p. 9). Health science curricula must reflect this focus and address interpersonal and systemic racism to heighten practitioner awareness and encourage institutional reforms.

This paper, which reports on early career midwives’ observations, experiences and responses to racism in maternity settings is part of a longitudinal investigation into the legacy of university initiatives to enhance midwives’ cultural capabilities. All participants took part in a larger study conducted in the period 2012–2014 while completing a midwifery program at a Western Australian university. One component of the study explored the impact of a new, first-year Indigenous health and cultures unit, learnings from a remote clinical placement offered to final-year students and Aboriginal students’ training experiences [26,30,31]. Findings from classroom observations, pre- and post-unit surveys and/or interviews highlighted a positive shift in attitudes in response to the unit but also drew attention to unresolved issues around race [26]. Another group interviewed had undertaken short clinical placements in remote community settings, an experience that was highly valued by students and offered opportunities for considerable cultural learning [31]. The interviews with Aboriginal students documented their struggles and successes as they pursued a career in midwifery [30].

The compulsory Indigenous unit was included in a new, interprofessional common first year for health science students introduced in 2012 and designed and taught in partnership with Aboriginal academics. Topics included family structures, past policies and practices, diversity in communities, and cultural beliefs related to health, pregnancy and birthing [26]. Personal stories which highlighted the impact of dispossession, cultural loss, removal from families, resilience and language revitalisation were presented using vodcasts. These were conducted and recorded by the Aboriginal coordinator and involved discussions (yarning) with Aboriginal community members. This technique provided a safe environment for participants to tell their stories and allowed students to encounter the lived experiences of a diverse group of Aboriginal people [26]. When the concepts of race and racism arose in classroom discussions, many differing views and experiences were aired, and expressions of considerable discomfort were noted by the lead researcher (R.D.T.) who had observed the class across a semester [26]. Discomfort was expressed verbally in classroom discussions when the experiences and attitudes of some students dismayed others. It was also recorded in interviews where participants could respond more openly about their feelings. While the discomfort and general unease surrounding the issue of racism often remained unresolved, it was hoped that both the unit content and the critical reflection that was encouraged would, over time, result in more informed and open responses to contentious issues raised [26].

The remote Ngaanyatjarra Lands placement was offered to selected midwifery students in the final year of their program. It commenced in 2010 and while some instruction in Aboriginal health had been received by students, it was instituted prior to the introduction of the new unit. Students who expressed interest were selected following an interview and undertook a 1–2 week placement under the supervision of the Aboriginal Community Controlled Health Service midwife [31]. Students worked in traditional and very isolated Aboriginal communities providing health services under supervision, mostly to the women. They learned about sensitive ‘women’s business’ (all aspects of reproduction, nurturing of children and ‘growing up’ strong Aboriginal women) and the difficulties confronted by women who had to re-locate for birthing. They also observed how health service delivery is impacted by the tyranny of distance and recognised the importance of culturally appropriate health promotion messages [31].

At the same time as the first study, three Aboriginal students were enrolled in or had just completed the direct-entry midwifery program which commenced in 2008. These low Aboriginal numbers reflect the national shortage of Aboriginal midwives who form only 1% of the midwifery labour force, well below population parity at 3.5% [32]. Each of these Aboriginal midwifery students was interviewed about their experiences undertaking the program. While their circumstances differed, none reported racism from those in the midwifery cohort although several examples of racism from within the wider interprofessional year group were provided. One student described her shock and dismay during her first lecture in a unit that included Aboriginal health to see a hand-written note by a class member which said “we’ve gotta do ‘coon’ health”. Given the offending nursing student left the lecture before the class ended, the behaviour was not addressed directly, although the lecturer was advised of the incident. The Aboriginal students had not undertaken the new Indigenous unit (as it was introduced later), nor had they completed the remote placement. All three completed the program and are practising midwives in Western Australia.

A series of papers has explored the immediate impact of these program initiatives on midwifery students who participated in the study, which was largely positive [26,27,30,31]. In the period 2019–2020, participants from each cohort were located and re-interviewed in a follow-up study, which aimed to determine the longer-term impact of Aboriginal program content and experiences on midwifery practice. The first paper focused on the legacy of program initiatives on the 14 non-Indigenous participants’ midwifery practice [33]. As part of the investigation, all 16 participants were asked about applying their knowledge in practice. This included whether they had observed or experienced casual or institutional racism in maternity settings and if so, how they had responded. Although an exploration of racism had not been an explicit research aim, the question related to working with Aboriginal women generated detailed responses from most participants, and observations related to racism form the focus of this paper. 

## 2. Materials and Methods

Follow-up data were gathered from 16 practicing midwives drawn from three separate but related cohorts.

- Cohort 1: Seven were former members of a class of 15 first-year midwifery students who completed the new Indigenous unit in 2012 and participated in the first study. When located in 2019, nine members of the cohort were practicing midwives, the inclusion criterion for the study. Seven of the nine midwives eligible to participate were re-interviewed; one declined the invitation; and one could not be located. The remaining members of the cohort had either not completed the program (4) or completed it but did not practice (2).
- Cohort 2: All seven were former midwifery students who completed a final-year clinical placement on the remote Ngaanyatjarra Lands in Western Australia in the period 2010–2013 and participated in the first study. All were practising midwives; each was located and re-interviewed.
- Cohort 3: Two of three former Aboriginal midwifery students were enrolled or had recently completed the program and were participants in the first study. All three were practising midwives; two were re-interviewed and one was contacted but unavailable.

The extensive networks of one of the authors (J.W.), a Clinical Midwife Consultant and former coordinator of the direct-entry midwifery program, were used to locate the study participants. All were provided with information about this study and invited to contact the lead researcher (R.D.T.) if willing to be interviewed. If interested, Participant Information and Consent Forms were emailed to potential interviewees and consent was acknowledged on the returned form or recorded at the commencement of the interview. Overall, a high response rate was achieved from the three cohorts (7/9; 7/7; and 2/3). This is likely attributable to the participants’ involvement in the first study and the rapport that existed between them and the lead researcher and clinical coordinator.

All efforts were made to limit the possibility of adverse effects on participants during the research process. In addition to informed consent and the option to leave this study at any time, other ethical considerations included de-identification of data, and minimisation of risk and harm to Aboriginal participants based on the National Health and Medical Research Council’s guidelines for Aboriginal health research [34]. All participants had previously been interviewed by the lead author (R.D.T.) and this further mitigated against harm due to the establishment of trust. Approval for this study was granted by the Human Research Ethics Office at the University of Western Australia in 2018 (ID: RA/4/20/4617).

All semi-structured interviews were conducted by the lead researcher (R.D.T.); fourteen were completed over the telephone and two face to face. Interviews, which lasted between 30 and 75 min were guided by an interview schedule prepared by the research team. Interviews were recorded with the participant’s consent and transcribed by the lead author. All audio files and completed transcripts were secured in the lead researcher’s password-protected computer. A number of transcripts were shared within the research team to enhance trustworthiness [35]. Thematic analysis involved the initial coding of transcripts, followed by multiple readings and the establishment of links between codes (axial coding). Complete immersion in the data commenced during the transcription process conducted by R.D.T. and continued as codes and the bracketing of key quotations were identified [36]. Categorisation and combining of codes led to the development of emerging themes, which were discerned deductively. These were subsequently refined as repeated patterns of meaning emerged. This labour-intensive, methodical and systematic process of analysis aimed to deliver trustworthy and credible findings, hallmarks of rigorous qualitative research [35].

## 3. Results

All participants (16) were female; their ages ranged from the late 20s to late 40s. Two participants identified as Aboriginal Australians. The majority had partners and children and two were on maternity leave at the time of interview. Twelve participants practiced in the Perth metropolitan area, two in regional/rural areas (Bunbury and Katanning), and one in Brisbane. Two had developing-country experience as volunteers, one in Uganda and the other in Mozambique, where she is currently based. All midwives, with the exception of one, worked in the public health care system, although one combined this with private practice and another with a research position at a local university.

### 3.1. Casual Racism: Midwives’ Perceptions of Prevalence

#### 3.1.1. Insights of Non-Indigenous Participants 

Thirteen of the fourteen non-Indigenous participants had witnessed casual racism in clinical settings. Most examples provided were directed towards Aboriginal women, but two midwives who practiced in multicultural settings identified widespread stereotyping of Indian patients. Disparaging comments were often heard in staff rooms but occasionally poor behaviour was observed in the presence of patients. One participant recalled the following incident while on a student placement:

*“A young Aboriginal girl came in in labour and this midwife became very authoritative, she didn’t offer her any options, but just told her what she had to do, I’ll never forget it. It was obviously because she was Aboriginal and she thought she could get away with it, I’m crying now thinking about it. I was the student observing this and she should have known better… it may have been nervousness on her part, and I don’t want to blame her but hope her practice has changed. *

*What I did notice was that the young Aboriginal girl tried to hide her face from her, sending a clear message, she created a physical barrier and eventually spat at her feet. The young midwife then marched out full of self-righteousness, how dare she?”*

It is noteworthy that the midwife who was the subject of the story and the participant were known to each other; while students, they had both completed the new Indigenous unit at the same university. The participant responded to the situation in the following way, recognising the value of what she had learned about supporting the patient and her family:

*“I played good cop. I pulled up my chair and sat with her family and tried to make them feel welcomed. I’d learned about the importance of trying to include the family. She didn’t want a cannula, I spoke softly to her and said she can put out her arm when she’s ready and eventually she did. I remember thinking, I have to fix this, build a bridge here...If she wants to bring her aunty in to help her make decisions about her care, then that’s up to her…we learned about that in the unit”.*

Most participants considered that they were now in a position to respond and challenge the observed behaviour although some acknowledged that it could be awkward to raise the issue with colleagues. *“Oh, I do, in a nice way, but I make it clear”.* Others commented *“Oh yes, I’m quite assertive so I’ll say something because I believe everyone deserves equal care regardless of background”* and *“when I was a grad, I didn’t say anything, except once, I said ‘that’s not very fair, you don’t know what’s she’s been through’. Now I’m more assertive and feel confident to address that”.*

#### 3.1.2. Insights of Aboriginal Participants

The Aboriginal participants viewed racism in maternity settings through a different lens; they had observed and experienced its impact first hand and were assertive in their efforts to identify it when it occurred. Both highlighted the damaging effects of stereotyping, and like their non-Indigenous peers, viewed stereotyping as widespread. The following experience had a profound effect on this midwife who was a student at the time.

*“It was night shift, and this young Aboriginal girl came up through Emergency. She was wheelchaired up, and she was basically ready to push. She was quite vocal, and they rushed her into a birth suite. All I could then hear was them yelling at her, and asking what drugs she has taken, they assumed she was on something and that’s why she couldn’t sit still and was jerking. And she kept saying she hadn’t taken anything…her Mum was there and said she’d only had Panadol, but they kept it up, ‘what have you taken’?*

*On reflection, that baby’s head was basically out and that’s why she couldn’t sit still…once she was on the bed, the baby was born. The whole experience was horrible for her. I was just a student but realised there was just no dignity at all for that girl. It’s always stayed with me.”*

When asked to identify the crux of the problem in this case, the participant pointed to “midwives who have been there for a long time, have a lot of power, sometimes think they know everything, are infallible…there is a silent acceptance that what you hear before you go into a room is right…but it may be just opinion, and it’s contagious”. The participant referred to how powerful this professional stereotyping is: “I truly think that the midwives don’t realise they are saying it…they become immune to their own stereotypes”.

Personal experiences of racism were also confronting for these participants. One recalled how she was discussing her background with an “older white nurse”. When she mentioned that her mother is Aboriginal and her father is white, the nurse replied: *“oh that’s why you’re doing so well”.* Despite being upset, she explained that her mother had a higher degree while her father left school at a young age. *“I saw it as an opportunity to educate…”* During the delivery of her sixth baby, she also had been asked whether all her children had the same father, commenting *“it made me feel really unsafe.”* The second Aboriginal participant constantly encountered “wide eyes” when she mentioned her background because of her pale skin. She also described using the response as an opportunity to educate those around her. *“I come up against racism every single day, but I realise that people aren’t always aware of what they are saying, so I’ll respond quite gently”.*

### 3.2. Institutional Racism: Midwives’ Perceptions of Prevalence

None of the non-Indigenous participants named incidents of institutional racism, although two acknowledged that its presence can be subtle. *“There are rules, there is so much in the system to say, hey, you ought to behave yourselves, hey you, none of that...but how the rules are enforced is another thing”.* Several examples were provided of organisational failures, which impacted on care given to Aboriginal birthing mothers. One related an *“over-zealous interpretation of the rules”* which prevented an elderly grandmother from the country being with her birthing granddaughter because her mother and sister were already with her; she slept overnight in the car. Another participant suggested that *“the way hospitals are run is not culturally friendly…I don’t think that’s institutional racism though”,* although another noted that *“I think our hospital does try to be as inclusive as possible...they have a flag and posters, there’s an Aboriginal garden and things like that”.*

Most incidents of racism cited by the Aboriginal participants were interpersonal in nature. However, both acknowledged the existence of institutional racism and recognised it when it occurred. One went so far as to say:

*“I truly believe that the hospital does do racial profiling. I’ve experienced it myself. It’s nothing that people even think about and I think that’s a reflection of white privilege. When you hand over, every race is mentioned except Caucasians, and everyone has an opinion or stereotype about how that woman is going to behave, before you even meet her. I’ve often wondered what would happen if I said, ‘oh there’s this white woman and she’s so non-compliant’...”*

Additional examples cited by participants of organisational failures which perpetuate stereotypes included the widespread assumption that Aboriginal health professionals are likely known by and can respond to any situation involving Aboriginal patients. One participant described an occasion where she was stopped in a corridor and asked to deal with a *“drug-addicted Aboriginal woman in Emergency…there was no confidentiality or consideration of how I might feel…so systemic racism is very real to me”*. That this happened frequently suggests widespread institutional misunderstandings about Aboriginal cultural diversity, interactions between family groups, and pressures placed on Aboriginal health professionals. In another incident, an Aboriginal student midwife on placement had described to the participant how she was introduced by a senior staff member as “part-Aboriginal”, a term linked to assimilation policies of the past. For a range of reasons, this student withdrew from her training *“it was all too difficult for her…and I’ve had that too, it’s continuous. I don’t give you permission to define me”.* These incidents were seen as indicative of an organisational culture in need of reform. Both Aboriginal midwives were keen advocates for organisational reform which better reflected Aboriginal women’s maternity care needs and Aboriginal health workforce issues; each used their unique positions and influence to promote initiatives and engage with community members.

### 3.3. Shifting Attitudes

Reflections on addressing casual racism in maternity settings were not directly sought from participants and responses were largely unprompted. The non-Indigenous midwives frequently referred back to the Indigenous unit or remote placement as powerful influences on their own heightened awareness of issues confronting Aboriginal women, despite racism not featuring prominently in their program. Midwives who had not encountered this content were considered less likely to reflect on their own attitudes. Several identified the need for more opportunities to expand knowledge and increase exposure to Aboriginal community-controlled health organisations, which offered a culturally secure model of service delivery. 

Aboriginal participants suggested a number of educational initiatives to address racism in maternity settings. One considered that *“stereotypes, racism and white privilege must be deconstructed, and stories of lived experiences presented”.* The inclusion of Aboriginal health professionals in classrooms and professional development sessions were considered essential. Exposure to Aboriginal Midwifery Group Practices was seen as an important way to reinforce unit content and allow students to see cultural security in action. It was also suggested that midwifery leaders should be more familiar with the Congress of Aboriginal and Torres Strait Islander Nurses and Midwives (CATSINaM), a very active professional body, which would *“change the perception that Aboriginal people need saving, need rescuing. We don’t need rescuing, we need allies”.* As one said, if non-Indigenous students and midwives went to CATSINaM conferences *“it would open their eyes...they’d be in the minority and the switch in power dynamics, well that can be profound, it has a really big role to play in changing perceptions”.* Furthermore, it was recognised that more Aboriginal midwives are required and that Aboriginal mentors are essential to both recruitment and retention of students and those practicing. School visits by community members also were seen as a way to inspire students about the midwifery profession and explain the pathways available to become a midwife.

## 4. Discussion

This study followed up participants who were initially interviewed and/or observed in a classroom setting in the period 2012–2014. At that time, most were midwifery students, but several had recently commenced practice. One of the aims of the earlier study was to explore receptivity to and learning acquired from a new, compulsory first-year Indigenous unit and a remote clinical placement completed by selected final-year students. In addition, information was gathered from the two Aboriginal midwifery students enrolled in the program and another who had recently graduated, exploring their experiences, motivations and progress through the program. The low number reflects the low proportion of Aboriginal people undertaking midwifery training. At the time, each of these participants reported very different experiences while completing the program. For one, it was transformative and built the confidence necessary for her to openly self-identify, for another it was marred by an experience of racism and on-going family challenges which had delayed the completion of her degree. The third Aboriginal participant, who was interviewed while practising, also completed her degree over an extended period of time due to extensive family responsibilities.

The follow-up study aimed to investigate the longer-term impact of the curriculum initiatives for working rurally and with Aboriginal people. As part of this, participants’ perspectives on the impact of their training included responding to Aboriginal women and hence their insights into racism in maternity settings were sought. The earlier study revealed that racism did not feature prominently in unit content, and when it was raised, had caused division and discomfort among students [26]. When professional experiences of racism were explored as part of the follow-up study, three main themes emerged from analysis of the interviews with the 16 participants: stereotyping as a manifestation of casual racism, discrepancies in recognising institutional racism and changing perceptions.

### 4.1. Stereotyping: The Manifestation of Casual Racism

A recent, large cross-sectional survey of Aboriginal Australians’ experiences of racism conducted in Victoria found that one-third of the 755 respondents had experienced racism in health care settings [37]. The authors noted that respondents “...most frequently reported being a target of racist names, jokes or teasing, or hearing comments that relied on stereotypes of Aboriginal Australians” [37]. These experiences were reported by just over half of respondents who had been subjected to racism in this setting. In maternity care settings, Brown and colleagues noted that fear of being stereotyped may act as a barrier to seeking care during pregnancy [22]. Furthermore, if Aboriginal women perceive their interactions with health professionals as disrespectful, their confidence in health services may be undermined and impact future utilisation [22]. Marriott and colleagues’ study of Aboriginal women’s birthing experiences also exposed the problem of stereotyping with one senior woman noting “there’s been times here at the hospital where some midwives have overstepped the mark well and truly, especially around comments and stereotyping” [21] (p. 399). This type of behaviour can make Aboriginal midwives as well as patients feel uncomfortable and potentially unsafe.

In this study, incidents of casual racism directed towards Aboriginal women and /or their family members in maternity settings were witnessed by 13 of the 14 participants who completed the Indigenous unit or remote placement; there were no obvious differences between the first two described cohorts. The Aboriginal midwives, however, had not only witnessed casual racism towards others, but suffered it themselves. Casual racism was consistently characterised by the participants as the stereotyping of behaviours of Aboriginal people, anticipating and judging a situation in advance of any interaction based on their pre-determined and largely negative expectations.

The non-Indigenous participants in this study appeared alert to casual racism and were aware that it was prevalent; for the most part, they reported being willing to call it out while also acknowledging that this can be awkward. Knowledge gained about Aboriginal history, health and cultures from program initiatives, especially from personal stories and interactions with community members, likely contributed to a heightened awareness of interpersonal racism in these two cohorts. The Aboriginal participants were more confident in their ability to address this type of racism, perhaps due to painful personal experiences, but also recognised the emotional labour involved in this process. Stereotypes abounded, with some behaviours incorrectly attributed to drug use and assumptions made about personal lives, partners, skin colour and hygiene. It was suggested that those harbouring negative views were largely oblivious to their impact. While these participants embraced an educative role when addressing racism, there is little doubt that potentially fraught interactions with colleagues and the constant need for vigilance and defending Aboriginal people and beliefs would exact a huge personal toll, both in the workplace and in the wider community.

One of the key objectives of the National Scheme’s Aboriginal and Torres Strait Islander Health and Cultural Safety Strategy is to encourage health practitioners to acknowledge and address individual racism. This requires a capacity to reflect on one’s own “biases, assumptions, stereotypes and prejudices” if culturally safe practice is to be achieved, and it is recommended that critical reflection be included in training programs [29].

### 4.2. Recognising Institutional Racism: An Impediment to Culturally Safe Care

Institutional racism has been widely recognised in an expanding body of literature as a significant contributor to poorer health outcomes for Indigenous Australians, affecting quality of care and access to services [7,13,38]. Durey and colleagues noted that institutional racism often starts at admission, where information gathered on Aboriginality, current address, next of kin and regular GP can be incorrectly recorded due to language and socio-cultural misunderstandings. Errors have significant consequences for patients, especially at time of discharge when follow up is required [38]. Further evidence of policies and practices that do not incorporate Aboriginal cultural values relates to restrictions on visitor numbers and reflects a profound misunderstanding of the centrality of the family, especially around birthing [21].

While alert to interpersonal racism, the non-Indigenous participants in this study did not specifically identify institutional racism, although several cited examples of systemic practices which appear to discriminate against Aboriginal patients on cultural grounds. These included two cases of family members being denied access to a birthing mother due to strict regulations on visitor numbers (on separate occasions a grandmother and a father slept in a car after being turned away, both had travelled from rural areas) and an acknowledgement that the hospital was not *“culturally friendly”.* Restrictions on visitor numbers, though universally applied, suggest inflexible organisational practices which could be more receptive to Aboriginal protocols surrounding childbirth. Efforts to create a more inclusive environment included the display of Aboriginal flags, posters and paintings and strict rules on staff behaviour, although several participants noted that institutional racism can be subtle, and rules were not always enforced. That most non-Indigenous participants accepted the policies and practices of their organisation likely reflects their position in the hierarchy and the influence of professional socialisation; this becomes the norm and encourages compliance, so rarely is the critical examination of processes rewarded [39]. Furthermore, the impact of institutional policies on vulnerable populations is not always fully understood.

Despite initiatives such as the flying of flags and display of Aboriginal artwork being considered as symbolic, both Aboriginal participants saw efforts to make Aboriginal culture more visible as significant. In fact, one of the midwives recently accepted a commission to complete a large painting for the maternity wing of a new Perth hospital. However, they readily identified institutional racism, with an example of racial profiling viewed as white privilege in action. While the behaviour described was interpersonal in nature, that it was not addressed was seen as an organisational failure. Other failures identified relate to narrow understandings of diversity within Aboriginal communities, issues of identity and unrealistic expectations of Aboriginal health professionals, all issues that would benefit from improved staff development and positive community exposure. In addition, they noted the slow progress to make policies and practices more culturally inclusive for Aboriginal women and to recognise their special needs during birthing, especially family support; these were regarded as systemic failures that must be addressed. What was highlighted was that discussions of racism in training programs must extend beyond the interpersonal domain and interrogate the potential for health professionals to engage in addressing systemic or institutional racism as part of caring and building respectful relationships with Aboriginal women. ‘Reconciliation Australia’s’ latest report describes this next step, where we are prepared to have uncomfortable conversations about racism with those within our sphere of influence and tackle racism head on, an action that moves from “safe to brave”, an action that accepts the risks involved and becomes the new agent of change [40].

### 4.3. Changing Perceptions

In this study, participants’ accounts of observed interactions between staff and Aboriginal women suggest that in some cases birthing mothers and family members were treated paternalistically rather than respecting their decision-making capabilities. Examples were provided of women being reprimanded for missed appointments, being disbelieved and generally considered incompetent. Such treatment is patronising at best and can lead to anxiety and disempowerment, it is especially challenging for women where English is a second language. One of Marriott and colleagues’ Aboriginal birthing women noted that “a white person can give off the signs, even if they’re totally a good person” [21] (p. 398). This behaviour,, although often unintended, can produce a sense of being undermined and diminished [21]. Similar experiences have been described by immigrant and refugee women who have suggested that more displays of kindness, access to information and involvement in decision making would go a long way towards improving interactions with maternity care providers [41].

Most participants in this study recognised the damaging effects of stereotyping, the importance of respectful communication and the need for attitude change among some midwives. Suggestions to improve Aboriginal women’s birthing experiences focused upon increased knowledge and exposure, and recognition of the power dynamics that existed in clinical encounters. In addition to enhancing midwives’ cultural capabilities, the Aboriginal participants also highlighted the need to increase Aboriginal health workforce participation.

However, the views of health professionals are shaped by social as well as professional norms. An examination of media portrayal of Indigenous Australian’s public health issues over a 12 month period found an abundance of negative stories (74%), with commonly used descriptors including “alcohol, child abuse, petrol sniffing, domestic violence, deaths in custody and crime” [42]. The authors suggested that these negative media portrayals not only perpetuate racist stereotypes but also affect the self-esteem of Indigenous Australians and risk being internalised. They identified a number of measures that have the potential to change attitudes and beliefs, including mass media campaigns and advocacy, and university programs that “target prejudice and false beliefs” [42]. While serious public health issues confront many communities, the deficit model fails to recognise individual and community achievements which highlight strengths, resilience and agency. The recent television series showcasing young Indigenous leaders by the ABC, Australia’s national broadcaster, suggests a welcome shift in emphasis but this approach is not widespread [43]. 

### 4.4. Limitations

While a very high rate of follow up was achieved, the students who completed the remote clinical placement were selected on interest and aptitude; this may have influenced the impact of the experience on their subsequent professional practice.

## 5. Conclusions

Racism in the health care sector undermines equitable service delivery, contributes to poorer health outcomes and has a detrimental effect on the recruitment and retention of the Aboriginal health care workforce. Culturally safe service provision requires health professionals to recognise and address personal prejudices and stereotypes, and acknowledge the determinants of Aboriginal health, including colonisation and systemic racism. These insights can be acquired from training programs and community placements if Aboriginal involvement is prioritised and Aboriginal voices are privileged.

Midwives’ insights into racism in maternity care settings, explored as part of a larger study into the legacy of program initiatives, provide encouraging evidence that the gains derived from exposure to well-designed Indigenous content in training programs and remote clinical placements were sustained and applied in the workplace up to seven years later. In particular, most non-Indigenous midwives recognised and were cognisant of the damaging effects of interpersonal racism and drew attention to poor behaviour among other staff when it was observed. However, they were less likely to recognise institutional racism even when citing examples of it. The Aboriginal midwives who had lived experience of both forms of racism were particularly alert to its corrosive effects and recommended a number of initiatives to change perceptions including switching the power dynamics by placing students in situations where they are in the cultural minority.

While the legacy of these curriculum initiatives has been largely positive, it is recommended that more attention be paid to the identification of institutional racism in educational programs and health care settings to heighten practitioner awareness and encourage organisational reforms. The concept of racism and its impact should be vertically integrated across programs to ensure that its complex manifestations are recognised and addressed. Until racism in all its guises is eliminated, Aboriginal people, whether birthing mothers, families or health professionals, risk feeling culturally unsafe and insecure in maternity care settings. Others exposed to racism are also impacted by its occurrence, so powerful, brave steps are needed to change the social norms in which racism occurs and goes unchallenged.

## Data Availability

Data are available only on request due to privacy considerations.
